# Archaea and Fungi of the Human Gut Microbiome: Correlations with Diet and Bacterial Residents

**DOI:** 10.1371/journal.pone.0066019

**Published:** 2013-06-17

**Authors:** Christian Hoffmann, Serena Dollive, Stephanie Grunberg, Jun Chen, Hongzhe Li, Gary D. Wu, James D. Lewis, Frederic D. Bushman

**Affiliations:** 1 Department of Microbiology, University of Pennsylvania School of Medicine, Philadelphia, Pennsylvania, United States of America; 2 Division of Gastroenterology, University of Pennsylvania School of Medicine, Philadelphia, Pennsylvania, United States of America; 3 Center for Clinical Epidemiology and Biostatistics, University of Pennsylvania School of Medicine, Philadelphia, Pennsylvania, United States of America; 4 Department of Biostatistics and Epidemiology, University of Pennsylvania School of Medicine, Philadelphia, Pennsylvania, United States of America; 5 Instituto de Ciências Biológicas, Universidade Federal de Goiás, Goiania, Goiás, Brazil; 6 Department of Biostatistics, Harvard School of Public Health, Boston, Massachusetts, United States of America; Oak Ridge National Lab, United States of America

## Abstract

Diet influences health as a source of nutrients and toxins, and by shaping the composition of resident microbial populations. Previous studies have begun to map out associations between diet and the bacteria and viruses of the human gut microbiome. Here we investigate associations of diet with fungal and archaeal populations, taking advantage of samples from 98 well-characterized individuals. Diet was quantified using inventories scoring both long-term and recent diet, and archaea and fungi were characterized by deep sequencing of marker genes in DNA purified from stool. For fungi, we found 66 genera, with generally mutually exclusive presence of either the phyla Ascomycota or Basiodiomycota. For archaea, *Methanobrevibacter* was the most prevalent genus, present in 30% of samples. Several other archaeal genera were detected in lower abundance and frequency. Myriad associations were detected for fungi and archaea with diet, with each other, and with bacterial lineages. *Methanobrevibacter* and *Candida* were positively associated with diets high in carbohydrates, but negatively with diets high in amino acids, protein, and fatty acids. A previous study emphasized that bacterial population structure was associated primarily with long-term diet, but high *Candida* abundance was most strongly associated with the recent consumption of carbohydrates. *Methobrevibacter* abundance was associated with both long term and recent consumption of carbohydrates. These results confirm earlier targeted studies and provide a host of new associations to consider in modeling the effects of diet on the gut microbiome and human health.

## Introduction

Humans live in association with immense populations of bacteria, viruses, fungi and archaea [1]–[8]. Many groups have now contributed surveys using deep sequencing to characterize these populations, revealing that the human microbiome differs radically at different body sites and among individuals [9]–[12]. Differences in body sites are associated with availability of nutrients, water, oxygen, and other site-specific features. The origin of differences between individuals is less clear, however, potentially reflecting distinct colonization early in life and different environmental exposures such as antibiotic use [13]–[15]. Another environmental exposure, ubiquitous but incompletely understood, is diet.

Recently, we reported correlations of long-term dietary patterns in 98 individuals and the bacterial lineages present in the gut microbiota [10]. Two genera, *Prevotella* and *Bacteroides*, were shown to have reciprocal patterns of abundance, paralleling several reports from others [16]–[18]. Abundant *Prevotella* correlated with consumption of carbohydrates, while abundant *Bacteroides* correlated with consumption of choline, fats, and amino acids. A short term controlled feeding study showed changes in the gut microbiota associated with the dietary interventions, but not a change in the overall structure of the bacterial community analyzed, supporting a role for long-term diet in determining the structure of the gut microbiome [10]. Another study recently reported that the diversity of the gut microbiota was linked with long-term diet, where a more diverse diet was correlated with an increased gut bacterial diversity [19].

Bacteria are abundant members of the gut microbiome, but not the only residents. Bacteriophage particles within the intestinal tract are present in potentially greater numbers than bacterial cells [20], [21]. Recently changes in bacteriophage communities in gut have been correlated with dietary interventions [5].

Archaea are also present in human gut, the most frequently occurring of which is *Methanobrevibacter smithii* [22]–[25], a methane producer from byproducts of bacterial fermentation [23]. Reported colonization rates by methanogenic archaea range from 25% to 95% of humans [26], [27]. In microbial ecosystems such as the human gut, when H_2_ accumulates due to bacterial catabolism, archaeal growth is stimulated associated with incorporation of H_2_ into methane [23]. Support for such syntrophy in the mammalian gut has been shown in a gnotobiotic mouse model, where co-colonization by *M. smithii* and *Bacteroides thetaiotaomicron* promoted increased growth of both species compared to mono-colonization [28].

Yeasts have been detected in human stool samples at least since 1917 [29], and by the mid 20th century their presence in the human intestine had been proposed to have a saprotrophic role [30]. Gut fungi may also be involved in pathogenic processes. Anti-*Saccharomyces* antibodies are detected in inflammatory bowel disease cohorts and are used as a predictor of disease progression [31], [32]. Recent work using a murine model has suggested that normally mutualistic or commensal fungi species may exacerbate intestinal inflammation in mice with sensitized genotypes [33]. In mice, over 14 fungal genera have been reported to be present within the mucus layer lining the intestinal epithelium [34]. Available data is likely incomplete, because of reliance mostly on culture-based methods. Recent reports using next generation sequencing also suggest diverse fungal communities in humans [35]–[37].

Based on the above, we hypothesized that the gut archaea and fungi are influenced by both diet and the other microorganisms present. Here we investigated these ideas in a cohort of 96 healthy individuals who were previously characterized for their bacteria/diet relationships [10]. Fungi were characterized by sequencing the Internal Transcribed Spacer region 1 (ITS1) of the rRNA locus and the archaea by sequencing a segment of the 16S rRNA gene. Short-term diet was characterized using a Recall questionnaire, and long-term diet characterized using a Food Frequency questionnaire. Analysis showed notable correlations of the three Domains of life with each other, and with dietary components–thus these data begin to specify potential multi-domain trophic interactions in the human gut microbiota.

## Results

### Samples for Analysis of the Relationship between Human Diet and Gut Microbial Populations

A total of 98 samples were collected from healthy volunteers, and sequences from 96 of these samples were used in this analysis after quality filtering. The archaeal and fungal components of the microbiota were assessed using the 16S rRNA gene and the ITS1 rRNA gene tags, respectively [38]–[40]. The bacterial population of these samples was characterized previously by 454 pyrosequencing of V1V2 segments of the 16S rRNA gene [10]. Volunteers were screened to be free of chronic gastrointestinal disease, cardiac disease, diabetes mellitus or immunodeficiency diseases, to have a normal bowel frequency (minimum once every 2 days, maximum 3 times per day), and body mass index (BMI) between 18.5 and 35.

### The Archaea of the Gut Microbiome

**Figure 1:**
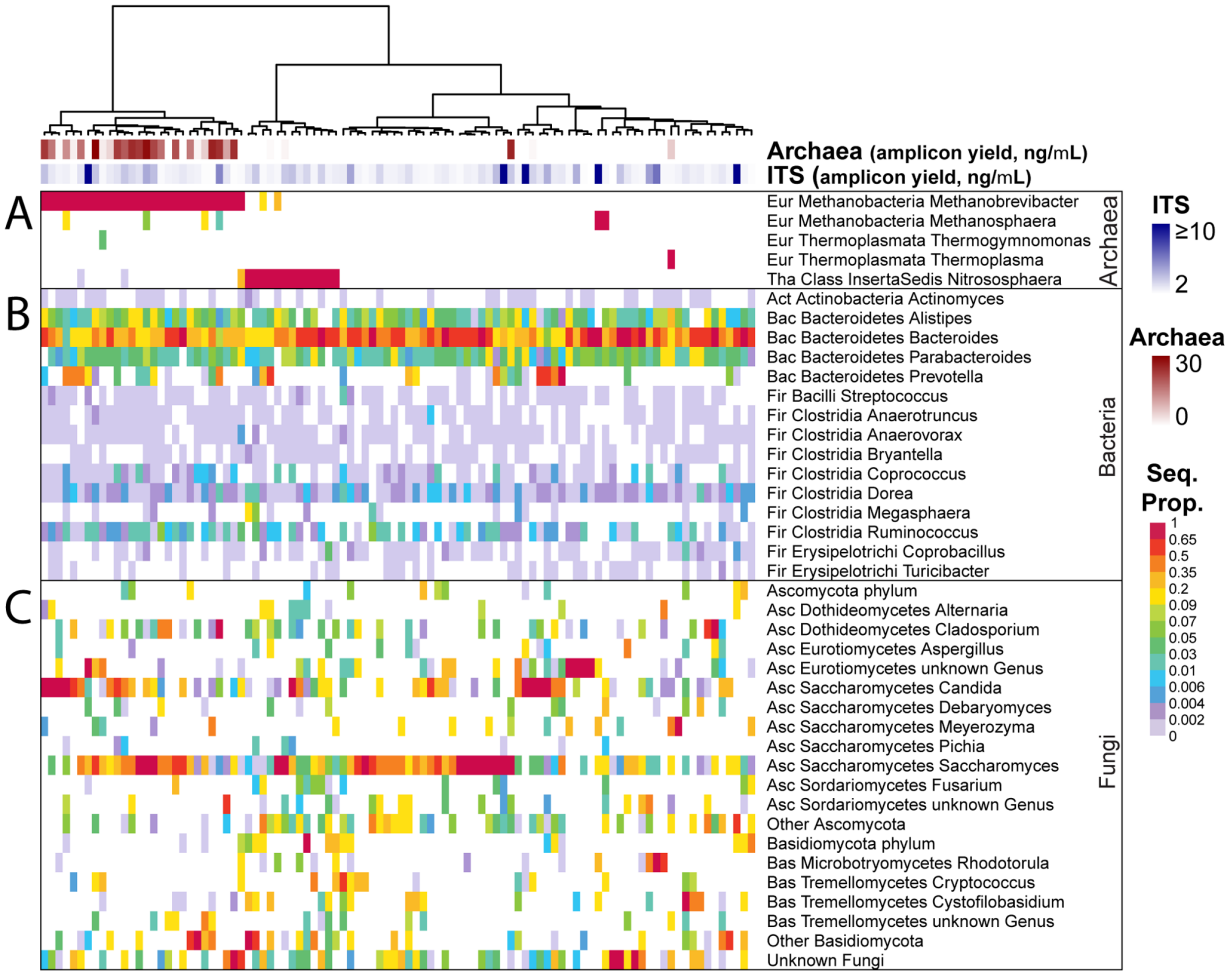
The archaeal and fungal components of the human gut microbiome. The heatmaps show the relative proportions of microbial lineages detected by pyrosequencing. The lineages are marked on the right, with Phylum (abbreviated), Class, and Genus. Archaeal genera are shown in (A), representative bacterial genera in (B), and fungal genera in (C). The top two rows show the DNA yield from PCR amplification reactions, which serves as a rough indicator of abundance. Proportions were calculated within each amplicon (archaeal 16S, bacterial 16S, or fungal ITS) for each sequencing study separately. The abbreviations for phyla were as follows (Eur: Euryarchaeota; Tha: Thaumarchaeota; Act: Actinobacteria; Bac: Bacteroidetes; Fir: Firmicutes; Asc: Ascomycota; Bas; Basidiomycota). Other Ascomycota and Other Basidiomycota are composed of genera which were detected in only one sample (see Table S7 and Figure S2 for a complete list of detected genera and their prevalence).

A total of 99,131 archaeal sequence reads were obtained, resulting in the detection of 5 genera (Figure 1). A total of 44 of the 96 samples analyzed were positive for at least one species. *Methanobrevibacter sp* was detected in 30 samples, and *Nitrososphaera sp* was detected in 16 samples (Figure 1A). The two genera were usually mutually exclusive, coexisting in only 6 samples.

The detection of *Nitrososphaera* was surprising and so was investigated further. Comparison of amplification efficiency suggested that the *Methanobrevibacter* when present was relatively abundant, while the *Nitrososphaera* was less abundant (Figure 1, top). *Nitrosophaera* was not detected after sequencing control amplifications with archaeal primers using products of blank DNA purifications as template (no positives out of eight tested). To validate the detection of *Nitrososphaera*, we used a nested PCR assay to detect the AmoA gene, which encodes the ammonia mono-oxyenase enzyme, and is distinctive for *Nitrososphaera*. We found an association between an AmoA positive PCR and *Nitrososphaera* detection in the samples (p = 0.014, Fisher’s exact test). The association, while significant, was not invariant, probably because of difficulties in detection due to the low level of *Nitrosophaera* in the samples, and possible presence of AmoA in other microbes or food materials. Inspection of published work showed detection of *Nitrosophaera* in metagenomic sequences from one of two individuals studied by [25], and in both 16S and metagenomic sequences in another cohort [41]. These data do not distinguish whether *Nitrosophaera* is replicating in the human gut or a transient present in food.

### The Fungi of the Gut Microbiome

The fungal sequencing effort yielded 332,659 sequence reads, resulting in detection of 66 genera and 13 additional lineages that could not be classified to the genus level (Figure 1 and Figure S1 and S2). Fungal sequences were detected in every sample analyzed. Only 12 fungal genera were detected in 9 or more samples (Figure 1B, Figure S2). The phyla Ascomycota and Basidiomycota were mostly inversely correlated (Figure S1). The most prevalent genus in this sample set was *Saccharomyces* (present in 89% of the samples), followed by *Candida* (57%) and *Cladosporium* (42%) (Figure S1).

### Co-occurrence Analysis using the Dice Index

Microbial communities in diverse settings have been shown to form syntrophic communities, in which metabolic waste products from one microbe provide nutrients for another. Thus an initial analysis of these samples was carried out to determine which microbes co-occur, as scored by the Dice index. For this, we only used data of relatively abundant genera (within Domain sample proportion of 0.01 or greater).

**Figure 2:**
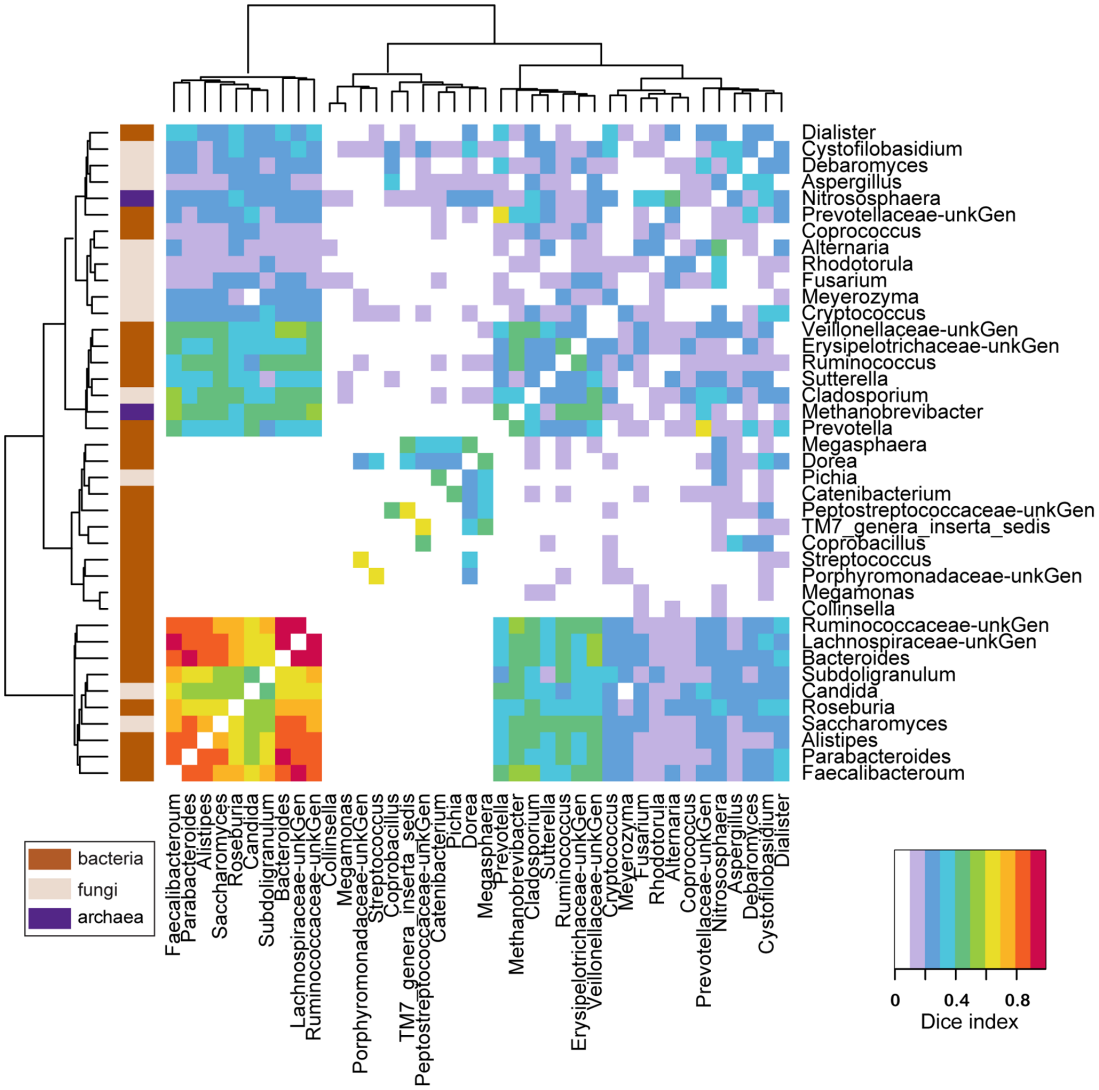
Analysis of co-occurrence among microbial lineages scored using the Dice index. Dice indexes across all genera pairs present at a proportion > = 0.01 are shown as a heatmap. Clustering was carried out using Ward’s criteria, based on the Euclidian distance between each genus pair using their Dice index across all other genera. Domain membership is color-coded on the left. Data are summarized in Table S9 and S10.

Numerous associations were detected. Figure 2 shows these interactions, incorporating data over all three Domains. Co-occurrence is indicated by the color code. Inspection of the figure shows several examples of co-occurrence involving a high proportion of samples (Figure 2, lower left corner, warmer colors). *Bacteroides* occurred commonly with *Parabacteroides*, *Lachnospiraceae*, and *Ruminococcaceae*. *Lachnospiraceae* occurred commonly with *Faecalibacterium* and *Ruminococcaceae* as well as *Bacteroides*. In contrast, co-occurrence of *Methanobrevibacter* and *Nitrososphaera* was low, as mentioned above.

*Candida* and *Saccharomyces* were both associated with the group containing the above bacteria. Several other fungi achieved levels sufficient for inclusion in the analysis, but showed less clearcut co-occurrence with other community members. *Methobrevibacter* showed modest levels of association with the above group of bacteria, but was similarly also associated with *Prevotella* and several other bacterial groups. *Nitrososphaera* showed only modest associations with other lineages. The frequent co-occurrence of some of these microorganisms suggests candidate interactions among gut microbes for further investigation.

### Covariation among Microbial Lineages

We next examined covariation among the three Domains, taking into account the relative abundance of each lineage in addition to presence-absence information. For the fungi and bacteria, multiple lineages were seen, and multiple different lineages were present in all samples, allowing use of correlation-based methods. However, for the archaea, only two lineages were detected with substantial frequency, *Methanobrevibacter* and *Nitrosophaera*, and these were mostly mutually exclusive. Thus for the archaea, samples were divided into three categories (containing one of the two archaea or no archaea), and co-occurring bacteria and fungi scored. For those few cases where both archaea were seen in a single sample, there was always a substantially greater abundance of one, so the sample was assigned based on the predominant lineage.

**Figure 3:**
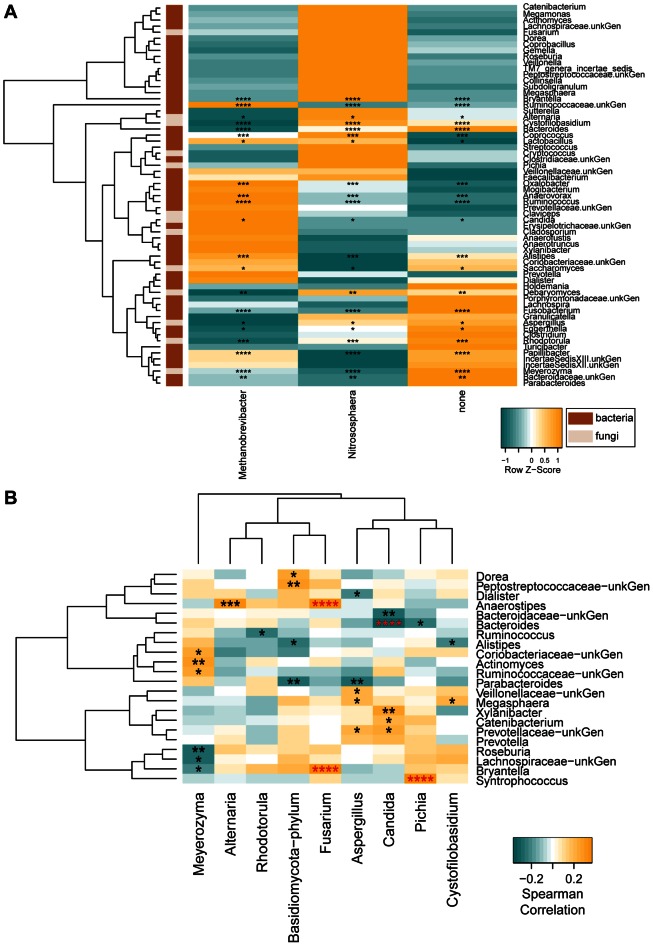
Inter-generic relationships. The heatmaps quantify the intergeneric relationships. (A) Normalized z-score of the bacterial and fungal proportions for samples grouped according to their archaeal status (*Methanobrevibacter* positive, *Nitrososphaera* positive, or archaea negative). Asterisks indicate Kruskall-Wallis significant comparisons after FDR adjustment (FDR of 25, 20, 15, and 10% are marked with 1, 2, 3 or 4 asterisks, respectively). Domain membership is color-coded on the left. (B) Spearman correlations between Fungi and Bacteria. Asterisks in red indicate FDR adjusted significant correlations (FDR 20%) and the remaining raw p-values are shown to illustrate general patterns within the data (p-values < = 0.05, 0.01, 0.005, 0.001 are marked with 1, 2, 3 or 4 asterisks, respectively).

As a first global test of association among the three Domains, we conducted a Permanova test using the newly developed Generalized Unifrac distance [42], which showed significance (Tables S1 and S2). To characterize the lineages involved, we used a non-parametric Kruskal-Wallis test to determine which bacterial and fungal genera co-varied with the archaeal categories (Figure 3A).

Several lineages co-varied with *Methanobrevibacter*, including the commonly encountered bacteria *Ruminococcus*, and rarer lineages such as *Oxalobacter* and *Papillibacter*. Of the fungi, *Candida* and *Saccharomyces* were both positively associated with *Methanobrevibacter*. Both fungal genera were negatively associated with *Nitrososphaera*.

The *Prevotella/Bacteriodes* ratio was implicated as important for the gut microbiome structure in previous studies [10], [16], [41], so we assessed correlations with fungal and archaeal taxa. We performed a PermanovaG test using the *Prevotella*/*Bacteroides* ratio and the generalized Unifrac matrices obtained from the fungal composition data. A significant relationship was observed (p = 0.0146) indicating a potential influence of the *Prevotella/Bacteriodes* ratio on fungi. A post-hoc test with the individual weighted and unweighted unifrac matrices showed that there was a significantly correlation between the *Prevotella/Bacteriodes* ratio and the weighted Unifrac distances (p = 0.0133), but not with the unweighted Unifrac matrix. Thus the *Prevotella*/*Bacteroides* ratio correlated with the amounts of fungi present, but not the types. For the archaea, *Bacteriodes* was significantly negatively correlated with *Methanobrevibacter*, but positively correlated with both *Nitrososphaera* and no archaea. *Prevotella* showed a reciprocal pattern, but it did not achieve significance, probably because of the lower numbers of *Prevotella*-positive samples in the data set.

We also used a PermanovaG test to determine whether the proportion of fungal phyla (Ascomycota or Basidiomycota) correlated with the gut bacterial lineages. Both Ascomycota and Basidiomycota were significantly correlated with the bacterial lineages (p = 0.0202 and p = 0.0037, respectively). A post-hoc Permanova test with the individual weighted and unweighted Unifrac matrices was significant only when using the weighted Unifrac matrix (p = 0.0205 and p = 0.004, for the Ascomycota and Basidiomycota, respectively). A targeted analysis of the Fungi (Figure 3B) showed a negative association of *Candida* with *Bacteriodes*. Together these results indicate that the types of fungal species in the gut were not correlated with the bacterial taxa present, but rather their relative proportions.

### Associations with Diet

We next investigated correlations between diet and the archaeal and fungal taxa. Dietary information was collected using diet inventories that scored usual (long-term) diet, and recent (short-term) diet. For each inventory, a clustering method was used to identify co-varying groups of dietary components, thereby reducing the number of variables and reducing penalties for multiple comparisons in the statistical analysis. We found that many of the clusters formed natural categories, such as carbohydrates, animal protein, and amino acids. Correlations between diet and bacteria have been reported previously for this data set [10] and were recapitulated here after using the pre-clustered dietary data (below). As a first step, Permanova tests were carried out using the nutrient cluster data and the weighted and unweighted Unifrac distance matrixes to determine whether the fungal and archaeal lineages were also associated with diet. Multiple significant associations were detected and are cataloged in Tables S3 and S4.

**Figure 4:**
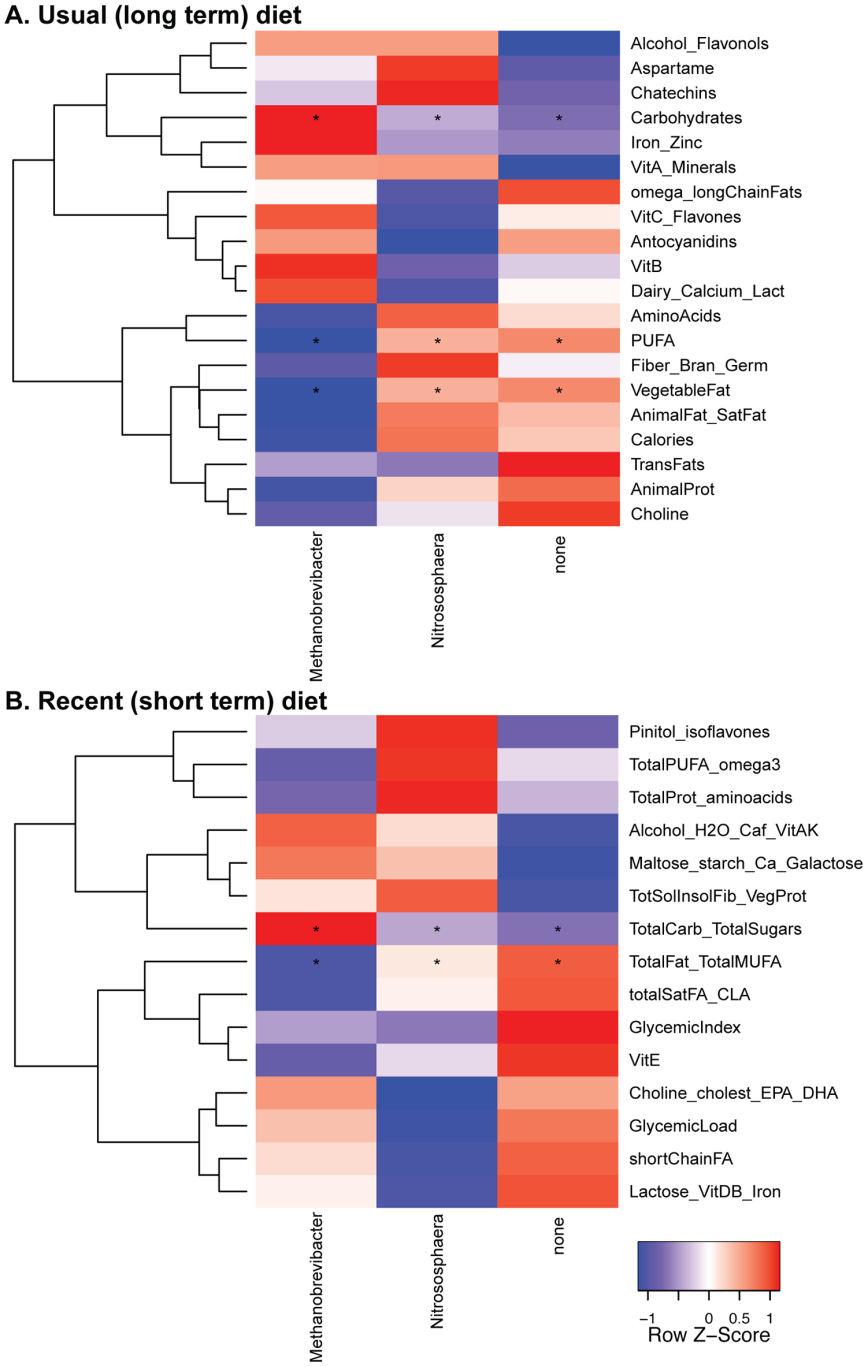
Archaea-Diet relationships. Heatmap of normalized average means for nutrient cluster measurements of the samples classified according to the dominant archaeal genus. Usual diet (A) and recent diet (B) relationships considered significant are marked with asterisks as described in Figure 2A.

Relationships between archaea and nutrients were explored using a Kruskall-Wallis test on the Permanova-selected nutrient cluster measurements. As above, samples were separated into archaea-negative, *Methanobrevibacter*-positive or *Nitrososphaera*-positive groups. A higher intake of carbohydrates was correlated with *Methanobrevibacter*-positive samples. This trend was observed in comparisons to both the long-term and short-term dietary data. For the long-term diet, samples with *Nitrososphaera* or no archaea were enriched for clusters representing vegetable fat and poly-unsaturated fats. Samples with no archaea were enriched for a cluster representing total fat and total mono-unsaturated fats in the recent diet data (Figure 4).

**Figure 5:**
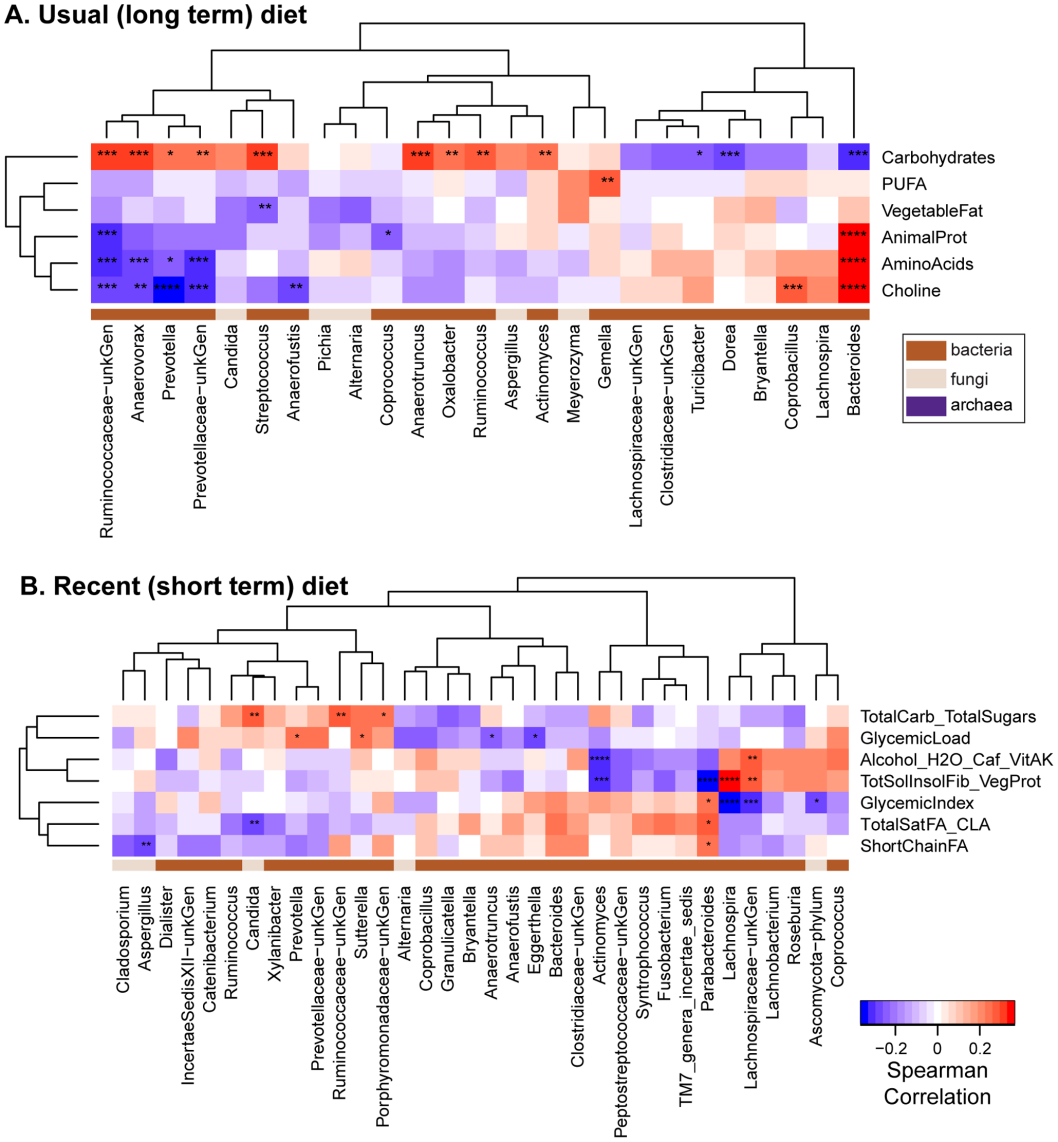
Fungi-Diet relationships. Heatmap of Spearman correlations between nutrient clusters and the bacterial and fungal genera detected in the dataset. Correlations which were considered significant using the Usual (A) and the Recent (B) diet data are marked with asterisks as in Figure 2A. Domain membership is color-coded on the bottom.

Fungal and bacterial proportions were also scored versus usual diet (Figure 5A) or recent diet (Figure 5B). Only diet categories with at least one significant association are shown. As reported previously, bacterial proportions were most strongly correlated with components of the long term diet–*Bacteriodes* was more abundant in individuals eating high levels of animal protein, amino acids, and fats, while *Prevotella* was higher among those eating higher proportions of carbohydrates. For fungi significant correlations were observed only with the recent diet inventory, differing from the observations with bacteria. In the recent diet data, *Candida* was positively correlated with carbohydrates and negatively with total saturated fatty acids. A trend in the same direction was seen for *Candida* in the usual long-term diet, but it did not achieve significance. *Aspergillus* was negatively correlated with short chain fatty acids in the recent diet data. No trends were seen with *Saccharomyces*. Thus, these data indicate that fungal abundance is particularly strongly associated with the composition of recently consumed foods.

## Discussion

Here we investigated the relationships of diet and the fungi and the archaea of the human intestinal microbiome. Previously we reported, for this same set of samples, that patterns in the bacterial part of the gut microbiome correlated with long-term diet. Here we characterized the sample set by sequencing marker genes of the archaeal rDNA 16S and the fungal ITS1 region. Many interactions among microorganisms and nutrients were identified. *Methanobrevibacter* and *Candida* were positively correlated with the ingestion of carbohydrates. This was most notable in short-term diet data for both groups, and in fact only achieved significance at all for *Candida* in the short-term diet data. These data support specific proposals for the interactions of members of the gut microbiome with dietary components and with each other.

We detected no fewer than 62 fungal genera and 184 species level OTUs, paralleling and extending a study of one subject, which also yielded a high number of fungal lineages [35]. Which of these lineages are true gut residents, and which are transients in food is unknown. Six individuals had sequences belonging to genus *Agaricus*, the white button mushroom, which is consumed as food. This genus was among those filtered out of the analysis due to low prevalence in the dataset, suggesting that fungal DNA in food may be mostly degraded during digestion. However, we cannot exclude the possibility that the high prevalence of *Saccharomyces* in the fungal data is due to the ingestion of yeast-containing foods such as bread and beer.

*Nitrososphaera*, which were encountered with unexpected frequency in our data, are different enough from other archaeal groups to be placed in their own phylum, the *Thaumarchaeota*. Members of the *Nitrososphaera* genus are able to oxidize ammonia and degrade urea, which presumably would also feed nitrogen into the gut microbial community. *Nitrososphaera* may have been previously underappreciated in microbiome studies due to its low abundance. Here, it was detected in 16% of the samples analyzed, though in low abundance, in a mutually exclusive pattern with *Methanobrevibacter*. The basis of possible antagonism between *Methanobrevibacter* and *Nitrososphaera* is unknown. *Nitrososphaera* also showed a positive association with the ingestion of proteins and amino acids, both for usual and recent diet. The correlation was not sufficient to survive correction for multiple comparisons, but may nevertheless indicate utilization of ammonia and or urea to meet their energy and carbon requirements. Alternatively, we cannot exclude the possibility that *Nitrososphaera* was ingested with foods, possibly associated with meats.

Previously, interactions among microbial lineages of the gut were proposed to separate human populations into “enterotypes”, leading to considerable controversy. Arumugam et al. [16] described three interacting networks of microbial lineages, centered on the presence of *Prevotella*, *Bacteriodes*, and *Ruminococcus*, together with other interacting taxa. However, subsequent work indicated that the most prominent feature of the data was an inverse correlation between the *Prevotella* and *Bacteroides* genera [41], [10]. Here we show a positive association of *Methanobrevibacter* and *Candida* with the *Prevotella* group, and each of these was further correlated with a diet high in carbohydrates. A negative correlation of *Methanobrevibacter* and *Bacteroides* was also observed, paralleling the original Arumugam et al. paper. However, we also observed a strong positive relationship between *Methanobrevibacter* and *Ruminococccus*, which were initially described to belong to distinct enterotypes. Thus our findings associate archaea and fungi with aspects of the enterotype concept.

**Figure 6:**
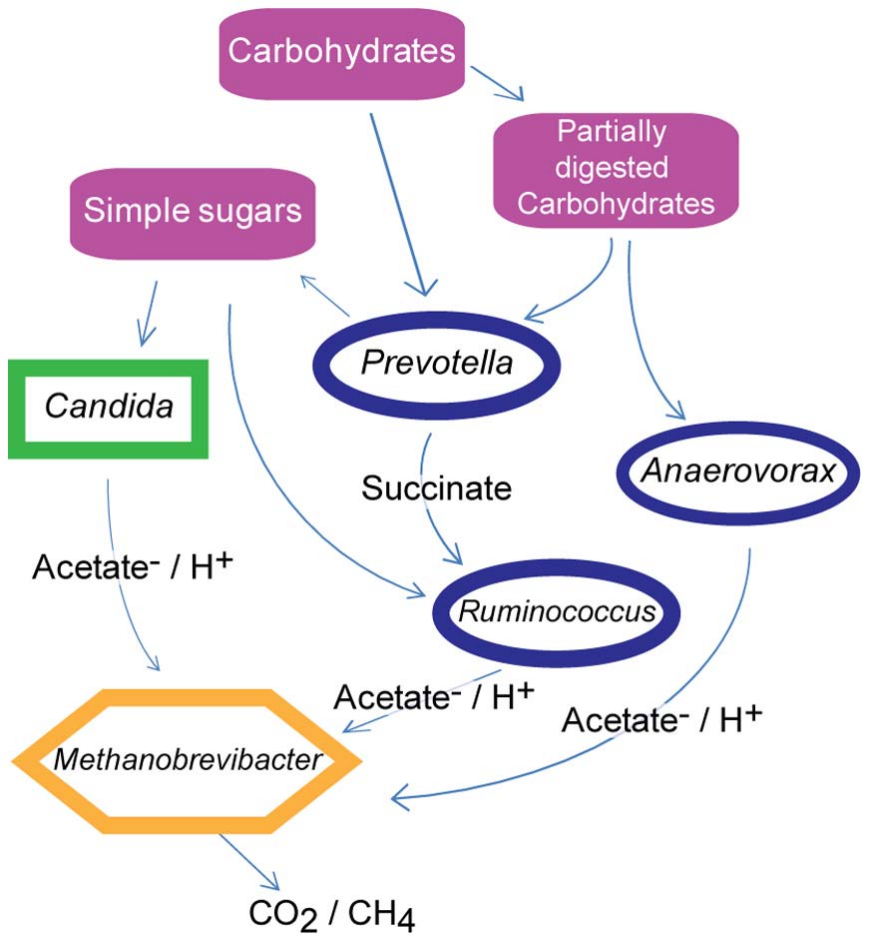
Possible syntrophic relationships in the human gut consistent with data reported in this study.

The intergeneric relationships described here, together with the nutrient correlations, support models for specific interactions among microbes in the human gut. An example of syntrophism has been previously described for *Ruminococcus* and methanogens, where the methanogens consume H_2_, allowing *Ruminococcus* to produce twice as many ATP molecules from the same amount of substrate [43]. One possible syntrophic guild specified in our data includes *Candida*, *Prevotella*, *Ruminococcus* and *Methanobrevibacter* (Figure 6). *Candida* is able to degrade starches, especially after pre-treatment with amylases [44] such as the human-encoded amylases present in the mouth and small intestine. Thus, in one model, *Candida* may assist in breaking down starch in carbohydrate rich foods, which in turn liberates simpler sugars to be fermented by bacteria such as *Prevotella* and *Ruminococcus.* Fermentation byproducts produced would then be consumed by *Methanobrevibacter* with the subsequent production of CO_2_ and/or CH_4_ [43]. Alternatively, *Prevotella* might degrade starch (pre-treated or not by human alpha amylases) and mannan containing polysaccharides from food to smaller poly- and monosaccharides [45]. *Prevotella* would then take up the smaller mono and polysaccharides, catabolizing them to produce succinate and other byproducts [46], [47]. All such hydrolysis is extra cellular, which would provide *Candida* with simpler sugars for fermentation (potentially down to acetate). *Ruminococcus* might then consume the succinate produced by *Prevotella* and produce H_2_ or acetate^−/^H^+^ for consumption by *Methanobrevibacter* [44], [48], [46].

In summary, the findings presented here provide a broader picture of the human gut microbiome, integrating its full diversity with human diet. The associations presented here, together with other work [49], [41], [13], allow the proposal of specific relationships between nutrition and microbial consortia within the human gut.

## Methods

In addition to the below, further information on methods can be found in the Supplemental Information, Figures S1–S3, and Tables S1–S10.

### Ethics Statement

The Institutional Review Board of the University of Pennsylvania approved all study protocols and all participants provided written informed consent, or assent in the case of minors. Legal guardians provided written informed consent for minors (protocol #810009).

### Samples

The samples described in [10] were used in this study. Bacterial 16S sequences data and diet data from [10] was used here. Briefly, healthy volunteers were screened to be free from any chronic gastrointestinal disease, cardiac disease, diabetes mellitus or immunodeficiency diseases, to have a normal bowel frequency (minimum once every 2 days, maximum 3 times per day), and body mass index (BMI) between 18.5 and 35. Demographic data was collect and analyzed as described previously for bacterial taxa ([10], Table S8). One stool sample was provided per subject and kept frozen at −80^o^C until processed for DNA extraction [10].

### 16S rDNA Gene, ITS1 Region and AmoA Gene PCR

Pyrosequencing was carried out using barcoded composite primers constructed as described in [50]. PCR reactions were carried out in triplicate using the Accuprime system (Invitrogen, Carlsbad, CA, USA). Each reaction contained 50 nanograms of DNA and 10 picoMol of each primer. Archaeal specific 16S rDNA primers and ITS1 fungal primers were adapted from the literature and used to amplify a rDNA 16S fragment; the final PCR cycling conditions were optimized to maximize specificity [51], [52], [38], [40],(Tables S5 and S6).

A nested PCR using specific primers for the AmoA gene was used to confirm the detection of *Nitrosophaera* sequences (Supplemental data. [53]). The nested PCR was performed to increase the sensitivity of the assay and improve the detection in these samples. Conventional PCR was not sufficiently sensitive to detect the 16S gene efficiently. PCR reactions were carried out using Invitrogen Accuprime. One µL of the total extracted DNA was used as template for the initial PCR. The nested PCR used 1 µL of PCR1 as template (Table S6). Blank extractions were used to control for environmental and reagent contamination. All PCR work was carried out in a laminar flow hood and all consumables and equipment were UV irradiated for a minimum of 30 minutes prior to use.

### Pyrosequencing

Amplified 16S rDNA and ITS1 fragments were purified using 1∶1 volume of Agencourt AmPure XP beads (Beckman-Colter, Brea, CA). The purified PCR products from the stool samples were pooled in equal amounts prior to pyrosequencing using Roche/454 Genome Sequencer Junior. DNA pools were separated by amplicon type. All samples were submitted for sequencing, even control samples for which no visible amplicon was observed in agarose gels. For such samples, 40 out 50 µL of the bead-purified PCR product was pooled with the other samples for sequencing.

### Sequence Analysis

Sequences obtained were decoded and quality controlled using the QIIME pipeline [54]. OUT’s were formed at 97% and 95% similarity for archaeal and fungal sequences respectively, and were considered for further analysis if they had a minimum of 5 sequences detected across all samples. Taxonomy was assigned to OTU representative sequences using the RDPclassifier [55] for archaeal sequences and BROCC [36] for fungal sequences. All taxonomy assignments were manually curated to check for accuracy and nomenclature using BLASTn against GenBank’s NR/NT database. For Archaea, the taxonomic assignment given by RDP to 2 out of the 12 archaeal OUT’s detected was corrected due to low coverage of those groups on RDP. For ITS, of the 290 OUT’s detected, 10 were missing mid-level (between phylum and genus) taxonomic information, which was filled in, and 5 yielded differing mid level taxonomies, of which only one of per genus was used. Two ITS OUT’s were automatically classified down to genus using BROCC, but had their taxonomy assignment brought up to Order level upon inspection of BLAST results (both OUT’s were present in one sample each). OTU sequence counts for each sample were aggregated at genus level. All downstream analysis was done at the Genus level using R unless otherwise noted. Genera were considered in the analysis if present in at least 9 out of the 96 available samples, and its absolute sequence count was equal to or greater than 10. For the ITS amplicon, samples were included in downstream analysis if they yielded at least 200 sequences. All novel sequence data was deposited at NCBI’s Sequence Read Archive under accession number SRP021021.

### Beta Diversity

Taxonomic information was used to obtain the Taxonomic Distance between each genus using the R package Ade4, version 1.5 [56], [57]. The taxonomic distance matrix was used as input to calculate Unifrac distances using the R package GUniFrac, version 1.0 [42].

### Inter-generic Relationships

Effects of bacteria on fungi, effects of the fungi on bacteria, and effects of archaea on bacteria and fungi were investigated using a Permanova test. Simulations of the effects of unequal variance in the different data sets compared indicated that differential variance was not a major confounder (Text S1). Within sample genus proportions were used to calculate Spearman correlations between bacterial genera and fungal genera. As only one or very few archaeal genera were detected in any sample, sequence proportions would be greatly skewed, invalidating any potential correlation results. Instead, samples were classified according to the archaea genera detected and bacterial and fungal proportions were used on as input for Kruskall-Wallis tests. P-values were considered significant using a FDR of 25%.

### Co-occurrence

The Dice index [58] was used to determine the co-occurrence of genera across the entire dataset. Genera were considered present in a sample if its sequence proportion was at least 0.01.

### Diet Analysis

Dietary information from [10] was used in this analysis. Usual diet was obtained using the Willett food frequency questionnaire [59]. Recent diet was obtained from 3 interviews recalling all consumed food on 3 days within the week preceding the sample acquisition (NHANES method, [60]). All interviews were carried out by trained nutritionists. Nutrient measurements across individuals obtained from the dietary questionnaires were used in a clustering procedure to reduce the number of comparisons to be made. First, Spearman correlations were calculated pairwise for all nutrient variables available and this correlation matrix was used as input in a clustering analysis, and 20 clusters (approximately 10% of the total number of nutrients available) were selected. Nutrients within each cluster were submitted to a Principal Component Analysis and the first principal component values were extracted and used as a surrogate dietary measurement (Nutrient Cluster Measurement). These nutrient cluster measurements were used in a Permanova analysis together with the taxonomy based, weighted and unweighted Unifrac distances calculated using the R packages Ade4 and GUniFrac. Clusters which were significant in the Permanova analysis were further used to calculate Spearman rank correlations using the proportion for each bacterial and fungal genus across all samples. The Nutrient Cluster Measurements for each sample were also classified according to their archaeal status and then submitted to a Kruskall-Wallis test to determine archaea/diet relationships. P-values with a False Discovery Rate of 25% or less were considered significant.

## Supporting Information

Figure S1 The Fungal phyla detected are shown as sequence proportions within each sample (A). A Spearman rank correlation for the proportions of Ascomycota versus Basidiomycota across the samples was 0.7456. Care should be taken when interpreting this correlation as the proportional nature of sequencing data naturally yields inverse correlations. The prevalence of each fungal genera detected across all samples is depicted in (B). Genera are grouped by their phylum affiliation and only genera sequences that could be assigned to the genus level are shown.
(TIF)

Figure S2 **Heatmap with all Fungal genera detected in the stool sample set used, and blank extraction controls.** Colors indicate relative proportion within each sample.
(TIF)

Figure S3 **Number of Fungal and Bacterial genera per sample.** Samples were classified as *Methanobrevibacter* positive, *Nitrososphaera* positive, or Archaea negative. Difference between groups was tested using a Kruskal-Wallis test, followed by a post hoc Dunn’s multiple comparison test. Asterisks indicate significant comparisons (p<0.001).
(TIF)

Table S1 **Permanova of Archaea effects on the Bacterial and Fungal parts of the microbiome.**
(XLSX)

Table S2 **Permanova of Fungal Phyla with Bacteria.**
(XLSX)

Table S3 **Permanova analysis of the association between usual dietary groups and bacterial, fungal, and archaeal lineages.**
(XLSX)

Table S4 **Permanova analysis of the association between recent dietary groups and bacterial, fungal, and archaeal lineages.**
(XLSX)

Table S5 **Primers used in this study.**
(XLSX)

Table S6 **PCR amplification conditions used in this study.**
(XLSX)

Table S7 **Number of reads for each genus, in each sample analyzed.**
(XLSX)

Table S8 **Permanova analysis of the association between demographic factors and bacterial, fungal, and archaeal lineages.**
(XLSX)

Table S9 **Co-occurring genera according to the calculated Dice index.**
(XLSX)

Table S10 **Positively co-varying genera.** Archaeal covariation represents the positive relationship detected in Figure 3A. Fungi/Bacterial relationships are the same as the positive relationships represented in Figure 3B.
(XLSX)

Text S1 Supporting information text.
(DOCX)
