# Supplementary figures and images for: Archaea and Fungi of the Human Gut Microbiome: Correlations with Diet and Bacterial Residents
Source: PLoS One. 2013 Jun 17;8(6):e66019. doi: 10.1371/journal.pone.0066019 (PMC3684604; doi:10.1371/journal.pone.0066019)

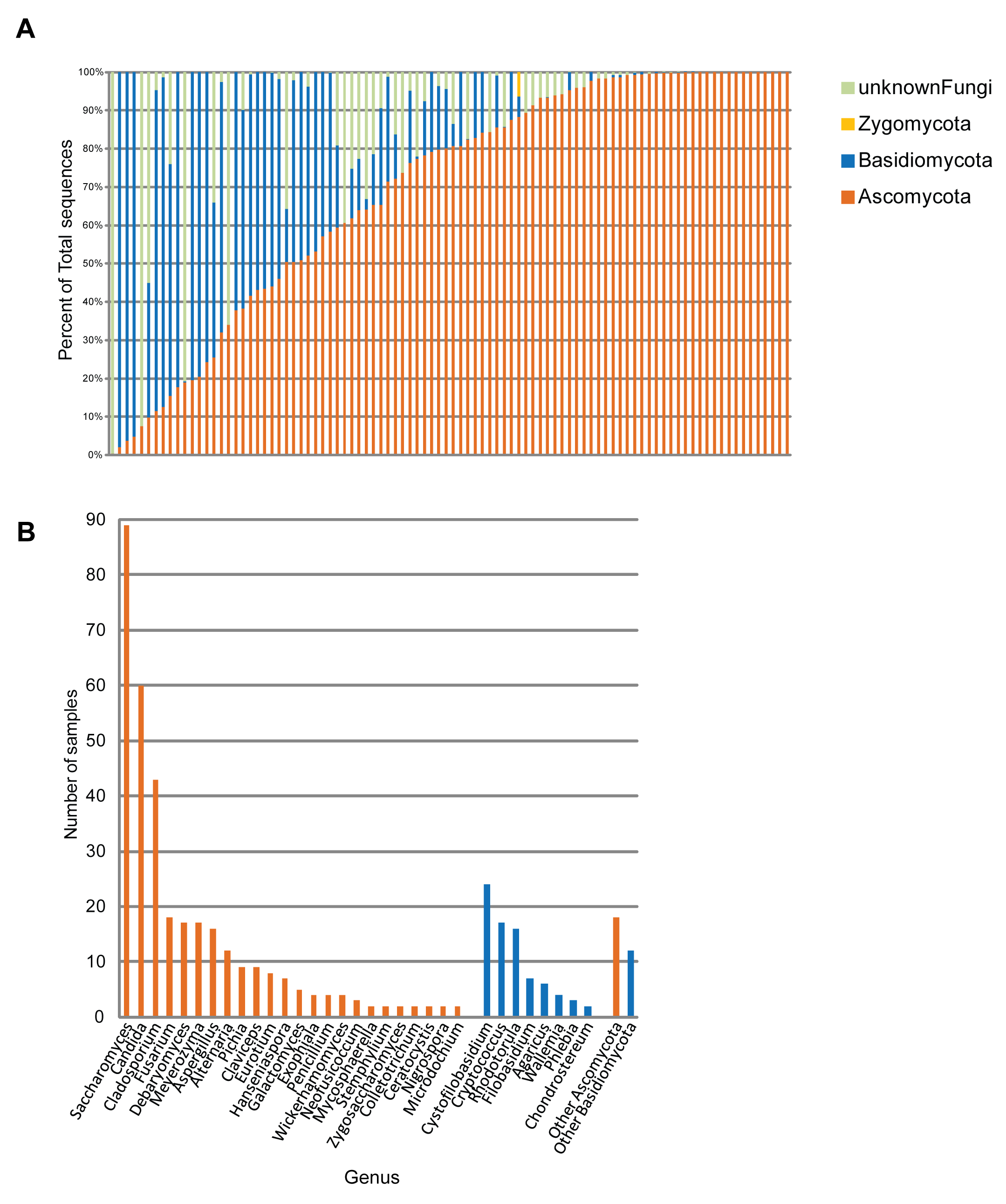

Supplement: Figure S1 — The Fungal phyla detected are shown as sequence proportions within each sample (A). A Spearman rank correlation for the proportions of Ascomycota versus Basidiomycota across the samples was 0.7456. Care should be taken when interpreting this correlation as the proportional nature of sequencing data naturally yields inverse correlations. The prevalence of each fungal genera detected across all samples is depicted in (B). Genera are grouped by their phylum affiliation and only genera sequences that could be assigned to the genus level are shown. (TIF) [file pone.0066019.s001.tif]

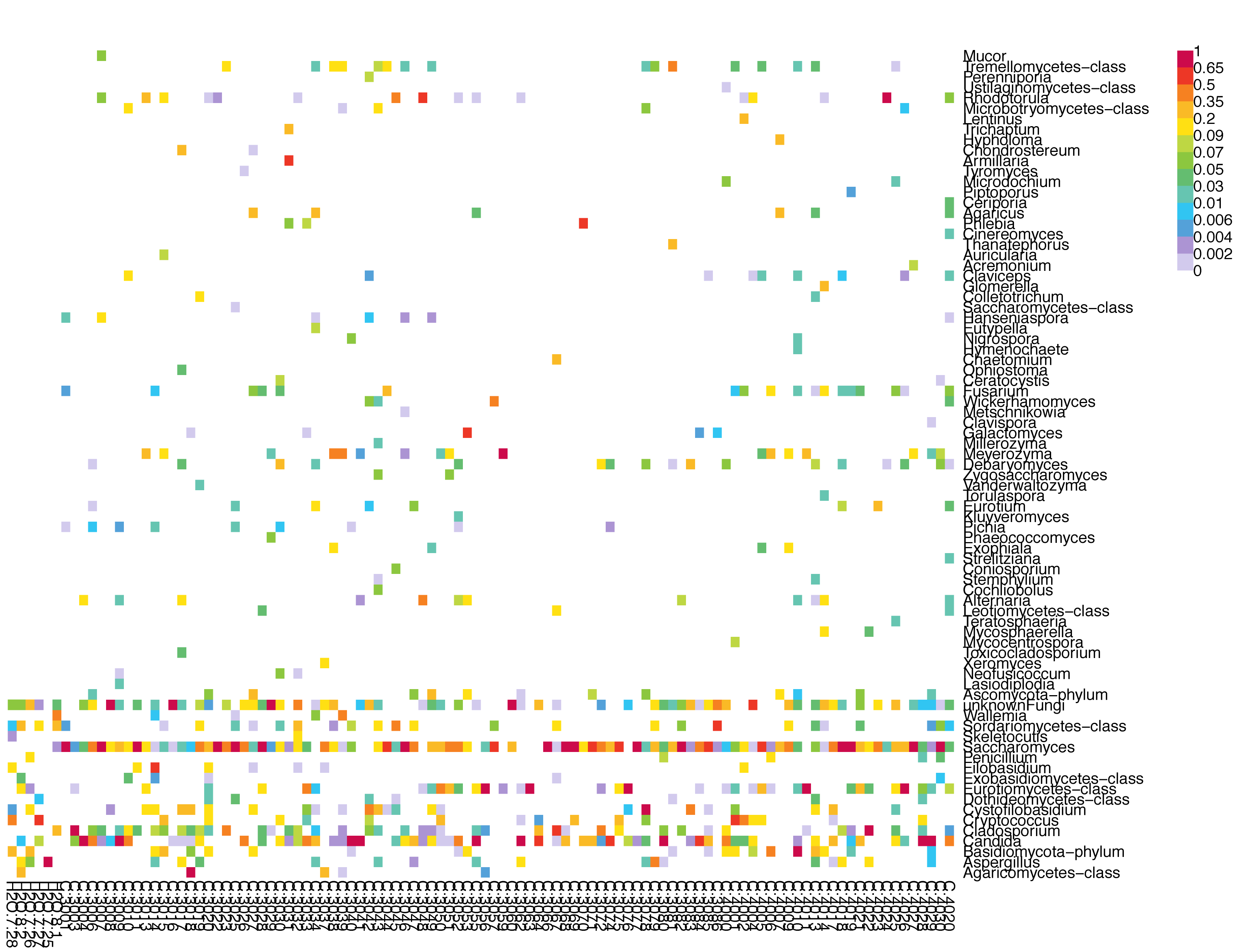

Supplement: Figure S2 — Heatmap with all Fungal genera detected in the stool sample set used, and blank extraction controls. Colors indicate relative proportion within each sample. (TIF) [file pone.0066019.s002.tif]

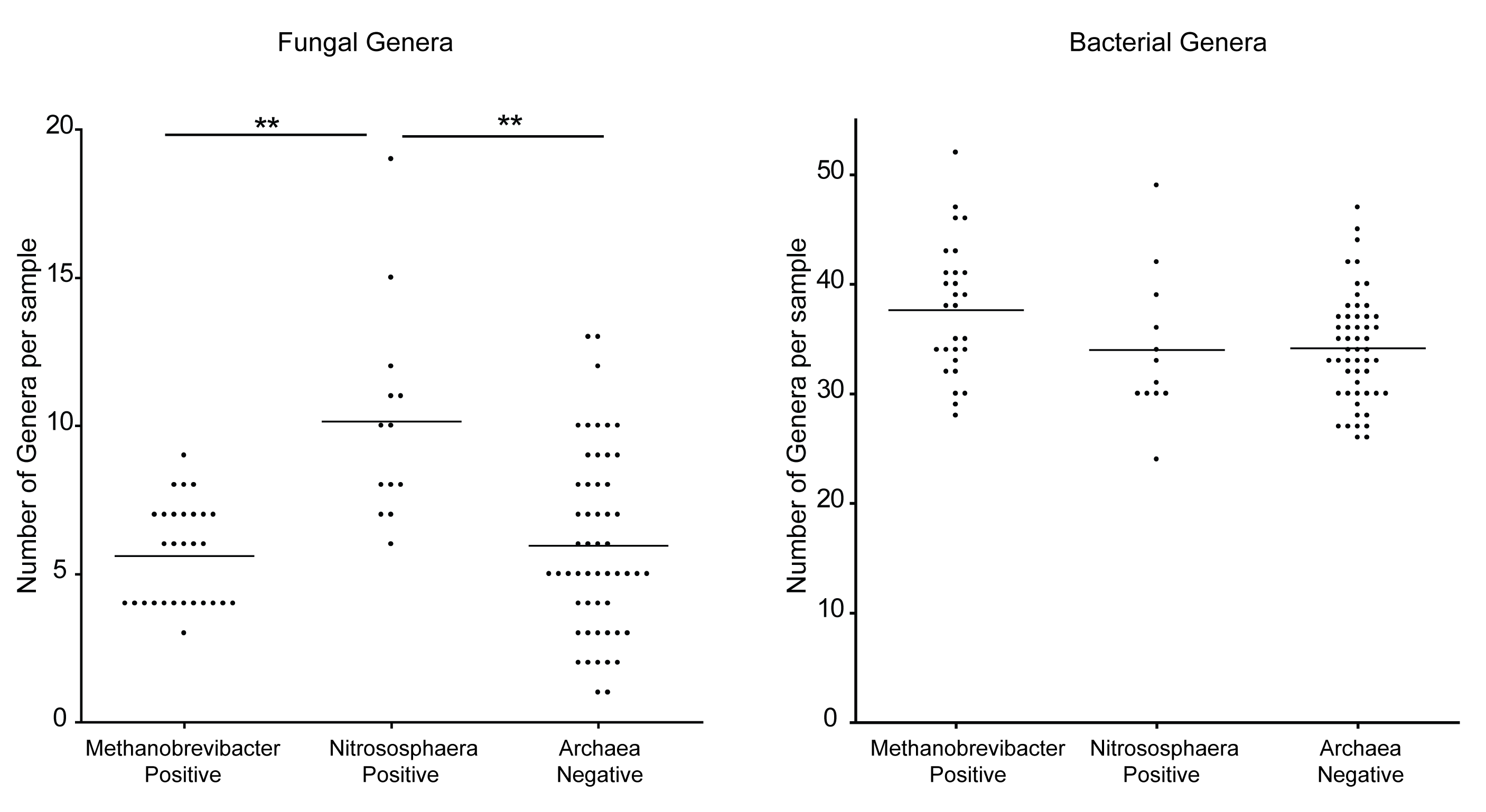

Supplement: Figure S3 — Number of Fungal and Bacterial genera per sample. Samples were classified as Methanobrevibacter positive, Nitrososphaera positive, or Archaea negative. Difference between groups was tested using a Kruskal-Wallis test, followed by a post hoc Dunn’s multiple comparison test. Asterisks indicate significant comparisons (p<0.001). (TIF) [file pone.0066019.s003.tif]
