# Supplementary material for: Archaea and Fungi of the Human Gut Microbiome: Correlations with Diet and Bacterial Residents
Source: PLoS One. 2013 Jun 17;8(6):e66019. doi: 10.1371/journal.pone.0066019 (PMC3684604; doi:10.1371/journal.pone.0066019)
Supplement: Text S1 — Supporting information text. (DOCX) [file pone.0066019.s014.docx]

**Supporting Information text for:**

**Archaea and Fungi of the Human Gut Microbiome: Correlations with Diet and Bacterial Residents**

Christian Hoffmann, Serena Dollive, Stephanie Grunberg, Gary D. Wu, James D. Lewis, and Frederic D. Bushman

**AmoA nested PCR analysis:**

The AmoA nested PCR was carried out for the 18 *Nitrososphaera* 16S positive samples as well as for 54 *Nitrososphaera* 16S negative samples. Seven out of 18 16S positive samples were also positive for the AmoA gene and 6 out of the 54 16S negative samples were positive for the AmoA gene. A Fisher's exact test was carried out using these nested PCR results versus 16S sequence data, and found to be correlated (Fisher's exact test, p=0.01378). No visible band was observed for any of the blank extraction controls (below).

**Negative controls for DNA extraction:**

Blank DNA extractions were carried out to control for reagent, consumable and environmental contamination during the procedure. In total, eight independent blank extractions controls, performed on 7 different days, were used.

For ITS1 region, triplicate PCR reactions were set up using 6 of these blank extractions, and no bands were observed in agarose gels. Nevertheless, triplicate reactions were pooled together and PCR purified using the AmPure XP beads (Beckman-Colter, Brea, CA), and eluted in a 50 μL volume of PCR grade water. 40 μL of the purified putative amplicon were sequenced as for fecal samples.

Blank extraction control sequences obtained were treated in the same manner as sample sequences. Twelve fungal genera and 7 unknown sequence groups at the genus level were detected in the extraction controls (supplemental figure 02). Only six of the known genera detected in the controls were also prevalent enough across the fecal samples to be included in the analysis. Three were not significantly correlated with other variables studied. One genus, *Cystofilobasidium*, could not be unambiguously assigned to the experimental samples and was not considered in further analysis.

**Associations with demographic factors:**

Demographic data was also recorded for the subjects tested, allowing correlation with abundance of microbial lineages using Permanova and weighted or unweighted UniFrac (Supplementary Table S8). Three demographic factors correlated with the fungal composition of the microbiota: the Unweighted analysis showed a relationship with Ethnicity and Smoking status, while the weighted analysis indicated a relationship with Ethnicity and Income. However, the number of individuals in for some of these groups was very small and care should be taken on interpreting these results. No associations were seen between demographic data and Archaeal lineages.

**Effects of unequal variance**.

Unequal variance among groups has the potential to confound statistical analysis. We therefore ran simulations on the PERMANOVA method used here, modeling the setting studied, and found little power for PERMANOVA to detect differences in variance, supporting the use of PERMANOVA in this work.

The R code used in the simulation is attached below.

The simulation settings were as follows: 2 groups, 50 samples each, 25 genera with majority of them being rare genera (p = 0.01),  200 reads/individuals for each sample. We use Dirichlet-Multinomial distribution to simulate the counts. The two groups have over dispersion 0.005 and 0.05 respectively (low and high spread).  By using this simulation strategy, on average we will see 20 genera in group A and only 10 genera in group B. The alpha diversity of the two groups are very different.  We then applied Permanova, recorded the p value and repeat the simulation 500 times. We calculated the power at type I error 0.05.  The power is 0.042 (= type I error), so very little power.

require(vegan)

require(dirmult)

nRep <- 500

nGen <- 25

nSam <- 50

nSeq <- 200

Pv <- numeric(nRep)

prop <- c(0.4, 0.2, 0.1, 0.05, 0.05, rep(0.01, 20))  # Common

abundance profile, majority genera are rare

for (i in 1:nRep) {

if (i %% 10 == 0) cat(".")

comm1 <- simPop(J=nSam, n=nSeq, K=nGen, pi=prop, theta=0.005)$data

# Low dispersion/spread, high alpha diversity

 comm2 <- simPop(J=nSam, n=nSeq, K=nGen, pi=prop, theta=0.05)$data

#Large dispersion/spread, low alpha diversity

X <- gl(2, nSam)

comm <- rbind(comm1, comm2)

comm <- comm / rowSums(comm)          # Convert to proportion as we

used in our analysis

Pv[i] <- adonis(dist(comm) ~ X)$aov.tab[1, 6]

}

sum(Pv <= 0.05) / nRep  # power
